# Pharmacological and Genetic Evidence for Gap Junctions as Potential New Insecticide Targets in the Yellow Fever Mosquito, *Aedes aegypti*

**DOI:** 10.1371/journal.pone.0137084

**Published:** 2015-09-01

**Authors:** Travis L. Calkins, Peter M. Piermarini

**Affiliations:** Department of Entomology, Ohio Agricultural Research and Development Center, The Ohio State University, Wooster, Ohio, United States of America; Virginia Tech, UNITED STATES

## Abstract

The yellow fever mosquito *Aedes aegypti* is an important vector of viral diseases that impact global health. Insecticides are typically used to manage mosquito populations, but the evolution of insecticide resistance is limiting their effectiveness. Thus, identifying new molecular and physiological targets in mosquitoes is needed to facilitate insecticide discovery and development. Here we test the hypothesis that gap junctions are valid molecular and physiological targets for new insecticides. Gap junctions are intercellular channels that mediate direct communication between neighboring cells and consist of evolutionarily distinct proteins in vertebrate (connexins) and invertebrate (innexins) animals. We show that the injection of pharmacological inhibitors of gap junctions (i.e., carbenoxolone, meclofenamic acid, or mefloquine) into the hemolymph of adult female mosquitoes elicits dose-dependent toxic effects, with mefloquine showing the greatest potency. In contrast, when applied topically to the cuticle, carbenoxolone was the only inhibitor to exhibit full efficacy. In vivo urine excretion assays demonstrate that both carbenoxolone and mefloquine inhibit the diuretic output of adult female mosquitoes, suggesting inhibition of excretory functions as part of their mechanism of action. When added to the rearing water of 1^st^ instar larvae, carbenoxolone and meclofenamic acid both elicit dose-dependent toxic effects, with meclofenamic acid showing the greatest potency. Injecting a double-stranded RNA cocktail against innexins into the hemolymph of adult female mosquitoes knock down whole-animal innexin mRNA expression and decreases survival of the mosquitoes. Taken together these data indicate that gap junctions may provide novel molecular and physiological targets for the development of insecticides.

## Introduction

The yellow fever mosquito, *Aedes aegypti*, is the most important vector of the viruses that cause yellow, dengue, and chikungunya fevers in humans. These diseases have spread around the tropical and subtropical world, facilitated by globalization of human societies and climate change [1]. In particular, chikungunya fever has most recently emerged from its native range in sub-Saharan Africa and Asia to Central America and the Caribbean in 2013–2014. As of 2014, locally acquired cases of chikungunya were reported in Florida [2,3].

Ideally, these mosquito-borne diseases could be prevented through the global use of safe, effective, and affordable vaccines. For yellow fever there is such a vaccine, however for chikungunya and dengue fevers there currently are no effective vaccines available [4]. An alternative strategy for controlling the spread of mosquito-borne diseases is to control populations of the mosquito vectors that transmit the pathogens. The primary control methods for reducing mosquito numbers are sanitation (cleaning and removing larval habitats from around homes) and using insecticides [5]. Although insecticides are effective at reducing mosquito populations, insecticide resistant populations have emerged because of the overuse of a few limited active compounds, such as pyrethroids [6]. The control of resistant populations of mosquitoes can be mitigated through a variety of techniques, including the development of new insecticides with novel modes of action, which begins with the identification of new insecticidal targets.

Gap junctions are potential molecular and physiological targets for the development of new insecticides. On the cellular level, gap junctions are intercellular channels that allow for the transport of small molecules and ions between adjacent cells [7]. On the molecular level, gap junctions are formed by two hemichannels from neighboring cells that dock with one another. Each hemichannel consists of six protein subunits, which are encoded by genes called connexins in vertebrates and innexins in invertebrates. The connexin and innexin proteins possess similar structural and functional features, but have evolved independently and thus their primary structures possess little similarity to one another [8,9].

In the *A*. *aegypti* genome, 6 genes encode innexins [10]; we have demonstrated that these genes are differentially expressed throughout the mosquito life cycle and in various tissues of adult mosquitoes [10,11]. In insects, innexins are known to play key roles in embryogenesis. For example, knockout of innexin 3 (*inx3*) in *Drosophila melanogaster* results in a failure of dorsal closure [12]. Moreover, in *Anopheles gambiae*, disruption of innexin 4 (a.k.a ‘*zero-population growth*’ or ‘*zpg*’) results in sterile males [13]. Innexins are also thought to be important in adult insect neuromuscular communication and renal function [10,14,15]. In summary, given the evolutionary distinct ancestry of innexins and connexins, as well as the consequences of innexin disruption on insect biology, we hypothesized that gap junctions may serve as valuable targets for insecticide development.

To test our hypothesis, we assessed the effects of gap junction inhibition on mosquito survival and/or physiology using pharmacological and genetic tools. In particular, we used three commercially available gap junction inhibitors (carbenoxolone, meclofenamic acid and mefloquine), which block vertebrate and invertebrate gap junctions formed by connexins and innexins, respectively [16–19]. Furthermore, we utilized RNA interference (RNAi) to knock down the expression of the 6 innexin mRNAs expressed in adult female *A*. *aegypti*.

We find that all 3 pharmacological inhibitors are toxic to adult female mosquitoes when injected into the hemolymph, and that carbenoxolone is effective when applied topically to the cuticle. Moreover, carbenoxolone and mefloquine decrease the diuretic capacity of adult female mosquitoes, suggesting disruption of Malpighian tubule function as a potential mechanism of action for these compounds. Also, carbenoxolone and meclofenamic acid kill 1^st^ instar larvae when added to their rearing water. Lastly, the knockdown of innexin mRNA levels in adult female mosquitoes decreases their survival over 11 days. Taken together, our results indicate that gap junctions are promising molecular and physiological targets for the development of novel insecticides to control mosquito vectors.

## Methods

### Mosquitoes

Eggs of *Aedes aegypti* were obtained through the Malaria Research and Reference Reagent Resource Center (MR4) as part of the BEI Resources Repository (Liverpool strain; LVP-IB12 F19, deposited by M.Q. Benedict). Mosquitoes were reared as described in Piermarini et al. [20] in an environmental chamber set at 28°C and 80% relative humidity with a 12 h:12 h light:dark cycle.

### Chemicals

Carbenoxolone, meclofenamic acid and mefloquine were obtained from Sigma-Aldrich (St. Louis, MO). All other chemicals were obtained from Thermo Fisher Scientific (Waltham, MA).

### Adult hemolymph injection assays

For direct hemolymph injection, carbenoxolone and meclofenamic acid were dissolved in HEPES buffered saline (HBS) as 100 mM stock solutions, whereas mefloquine was dissolved into 100% dimethyl sulfoxide (DMSO). Before injection, the inhibitors were diluted to their desired concentrations in HBS. The HBS consisted of 11.9 mM HEPES, 137 mM NaCl, and 2.7 mM KCl; the pH was adjusted to 7.45 using NaOH. For dilutions of carbenoxolone and meclofenamic acid, DMSO was added to the HBS at a final concentration of 11% to match that found in dilutions of mefloquine.

Adult female mosquitoes (3–10 days post-eclosion) were immobilized on ice prior to injecting their hemolymph with 69 nl of an inhibitor using a Nanoject II microinjector (Drummond Scientific Company, Broomall, PA). For a given dose of a compound, ten mosquitoes were injected and transferred to small cages (10 mosquitoes per cage) with access to a 10% sucrose solution. The cages were returned to the rearing chamber and the efficacy of a dose was assessed 24 h later, as described in Raphemot et al. [21]. In brief, the efficacy was measured as the percentage of treated mosquitoes in a cage that were incapacitated by 24 h; i.e., the collective percentage of mosquitoes that were flightless or dead [21]. A total of five to ten independent replicates were performed for each dose of each inhibitor.

### Adult topical assays

For topical application, all inhibitors were dissolved directly at their desired concentrations in 75% ethanol/25% H_2_O. Adult female mosquitoes (3–10 days post-eclosion) were immobilized on ice and a Hamilton repeating dispenser (Hamilton Company, Reno, Nevada) was used to apply 500 nl of an inhibitor to the thorax of each mosquito. For a given dose of compound, ten mosquitoes were treated and transferred to small cages (10 mosquitoes per cage) with access to a 10% sucrose solution. The cages were returned to the rearing chamber and the efficacy of a dose was assessed after 24 h, as described above in ‘*Adult hemolymph injection assays’*. A total of four to eight independent replicates were performed for each dose of each inhibitor.

### Adult excretion assay

The diuretic capacity of adult female mosquitoes was assessed using an established protocol [21]. In brief, for a given treatment, 3 mosquitoes (3–10 days post eclosion) were immobilized on ice and injected with 900 nl of HBS into their hemolymph using a Nanoject II microinjector (Drummond Scientific). After injection, the mosquitoes were placed into a graduated, packed-cell volume tube (MidSci, St. Louis, MO) for two hours at 28°C to allow them to excrete. After two hours, the mosquitoes were removed from the tube, which was then centrifuged at 17,000 g to allow for the excreted volume to be measured visually via the graduated column at the bottom of the tube. At minimum, 6 replicates (3 mosquitoes per replicate) were performed for each treatment. All mosquitoes were confirmed to be alive at the end of the two hours. Mosquitoes that were not injected with HBS served as controls.

The composition of the HBS was similar to that described above in ‘*Adult hemolymph injection assays*’ except that NaOH and DMSO were reduced to 1 mM and 2%, respectively. For a given experiment, one of the following inhibitors was added to the HBS at the indicated concentrations: carbenoxolone (1.34 mM), mefloquine (0.5 mM), and meclofenamic acid (1.53 mM).

### Larval assays

The toxicity of the inhibitors on 1^st^ instar larvae was assessed using the protocol of Pridgeon et al. [22]. In brief, eggs were hatched in dH_2_O under vacuum at room temperature for 2 h in a 250 ml beaker. The beaker was transferred to a rearing chamber (28°C) and 1^st^ instar larvae were collected 24 h later. For a given treatment, 5 larvae were transferred to the well of a 24 well Falcon MULTIWELL plate (Becton Dickinson Labware, Franklin Lakes, NJ) containing 995 μl of H_2_O with a gap junction inhibitor and 5 μl of food solution. The food solution consisted of 0.013 g of ground Tetramin fish food (Tetra United Pet Group, Blacksburg, VA) suspended in 1 ml of dH_2_O. Stock solutions of the inhibitors were dissolved in dH_2_O and diluted within wells to achieve the desired concentrations (1 ml total volume per well). Plates were returned to rearing conditions and after 24 hours the larvae were assessed. The efficacy of a concentration was measured as the percentage of treated larvae in a well that were dead by 24 h. Larvae were considered dead if they did not move when disturbed with a 10 μl pipette tip.

### dsRNA synthesis and injection

dsRNAs were synthesized from DNA templates. To generate the DNA templates, primers were designed for each innexin cDNA using Primer3 software [23] to amplify a 300–500 base pair product (Table 1). The nucleotide sequences of the primers and expected PCR products were subjected to a Basic Local Alignment Search Tool (BLAST) against the *A*. *aegypti* genome (Vectorbase.org) to ensure specificity. A T7 promoter sequence (TAATACGACTCACTATAGGGAGA) was added to the 5’ end of each forward and reverse primer to allow for dsRNA synthesis. The DNA templates were generated via PCR, which consisted of mixing 0.5 μl plasmid DNA (from previously cloned innexin cDNAs), 1 μl forward and reverse primer mix (10 μM each) and 23.5 μl Platinum Taq DNA polymerase High Fidelity (Thermo-Fisher). The mixture was subjected to a thermocycling protocol consisting of an initial denaturation at 95°C (2 min) followed by 30 cycles of 95°C (1 min), 60°C (30 sec), 72°C (1 min); the protocol ended with an elongation step at 72°C (5 min). The identities of the various DNA templates generated by PCR were verified by agarose gel (1%) electrophoresis and Sanger DNA sequencing at the Molecular and Cellular Imaging Center (MCIC) of the Ohio Agricultural Research and Development Center (OARDC) of the Ohio State University (Wooster, OH).

**Table 1:** dsRNA template synthesis primers. Each primer set consists of an innexin specific region for amplification of the target gene from plasmid, and the T7 promoter sequence (TAATACGACTCACTATAGGGAGA).

	dsRNA Template Forward	dsRNA Template Reverse
**Inx1**	TAATACGACTCACTATAGGGAGAGCGAAGCTGCAGAAGCTATT	TAATACGACTCACTATAGGGAGAAAATGTTTTGTCGAGGTTCATGT
**Inx2**	TAATACGACTCACTATAGGGAGATTTGGCGTTTGAAAAGTGTG	TAATACGACTCACTATAGGGAGAATACTCCCGGCTGAGCAATA
**Inx3**	TAATACGACTCACTATAGGGAGACGACGGTGACAGATTGACTAG	TAATACGACTCACTATAGGGAGAGTTCGCTCCTGGTTGTACTC
**Inx4**	TAATACGACTCACTATAGGGAGACATTCCTGTTCTCGTTCCCC	TAATACGACTCACTATAGGGAGAAAGGCACAGGGCATCAAAGT
**Inx7**	TAATACGACTCACTATAGGGAGACAGGGACAATCCAAAAGCATG	TAATACGACTCACTATAGGGAGATCAGTTTCGTCAGCCTCATC
**Inx8**	TAATACGACTCACTATAGGGAGATTCTGACGATACTGACGACGTT	TAATACGACTCACTATAGGGAGATCATGCATCCTGTATTTCACCT
**eGFP**	TAATACGACTCACTATAGGGACGTAAACGGCCACAAGTT	TAATACGACTCACTATAGGGTTGGGGTCTTTGCTCAGG

Template DNA was then used in the T7 MEGAscript dsRNA synthesis kit (Thermo-Fisher Scientific) following the manufacturer’s protocol (20 μl total reaction Volume). The resulting dsRNA was resuspended in nuclease free water and its concentration was measured on a Nanodrop 2000 spectrophotometer (Thermo Scientific). The dsRNA for each innexin was diluted to approximately 4 μg/μl, aliquoted, and stored at -80°C to avoid repeated freeze-thaw degradation.

On the day of an experiment, all six innexin dsRNAs were diluted to 2 μg/μl in a PBS solution (137 mM NaCl, 2.7 mM KCl and 11.9 mM phosphates; pH 7.5). Before injection into a mosquito, equal volumes of each innexin dsRNA were mixed together to form an innexin dsRNA ‘cocktail’, resulting in a final concentration of ~333 ng/μl for each dsRNA. Adult female mosquitoes were anesthetized on ice and their hemolymph was injected with either 1 μl of the innexin dsRNA cocktail or a negative control dsRNA against enhanced green fluorescent protein (eGFP; 2 μg/μl) using a Nanoject II injector (Drummond Scientific). For the eGFP and innexin dsRNAs, a total of 30 mosquitoes were injected per replicate (6 mosquitoes were dedicated for qPCR analysis and 24 were dedicated for survival assays). After injection all mosquitoes were placed in small cages and returned to rearing conditions. Four biological replicates were performed for knockdown analysis and three biological replicates for the survival assay.

### Phenotype assessment and qPCR

Mosquitoes injected with eGFP or innexin dsRNA were checked at 24 h intervals for 11 days and the number of surviving mosquitoes was recorded. Real time quantitative PCR (qPCR) was utilized to determine knockdown on days 3, 7 and 11 post-injection. RNA extraction and cDNA synthesis were performed as described in Calkins et al. [11]. Primers for qPCR were designed against the six innexins and ribosomal protein S7 (RPS7; a reference gene) using Primer3 software [23] to amplify a 90–110 base pair product (Table 2). Specificity of the resulting PCR products was confirmed using a melt curve analysis and Sanger sequencing (MCIC, OARDC).

**Table 2:** qPCR primer pairs. Each set of primers was selected for innexin specificity and determined specific through melt curve analysis and sequencing (MCIC, OARDC).

	qPCR Forward	qPCR Reverse
**Inx1**	CACCGATAGTGCCGTATTCC	CCGACATATTGTGTGGCAGT
**Inx2**	GGAGATCCTATGGCACGAGT	ACGGTAGCACACAGAGTCCA
**Inx3**	TCGTTCGGTTACTTCATCTGC	GCGATTCTCCTGATCCATGTC
**Inx4**	TTCTGTTGGACACTGGGAAC	CCATGTGCGTTCCTATTTCG
**Inx7**	TGGGTCCCGTTTGTGTTATT	CCATACGAAGACCATCCACA
**Inx8**	GACTGCGTTCACACGAAAGA	GGGTACTTCGCTACCGACTTT
**RPS7**	CTTTGATGTGCGAGTGAACAC	CATCTCCAACTCCAGGATAGC

For a given sample, each qPCR consisted of three technical replicates of 10 μl reactions each consisting of 5 μl of GoTaq Master Mix, 40 ng cDNA, 400 nM forward and reverse primers, and nuclease free water. The reactions took place in 96-well unskirted, low profile plates (Bio-Rad Laboratories, Hercules, CA), sealed with TempPlate RT optical film (USA Scientific). qPCR was performed using a Bio-Rad C1000 thermocycler and CFX96 real time system (Bio-Rad Laboratories). The thermocycler used the following protocol: an initial denaturation of 95°C (3 min) followed by 39 cycles of 95°C (10 sec) and 58°C (30 sec), ending with a melt curve cycle.

### Data analysis and statistics

GraphPad Prism 6 software (GraphPad Software Inc., La Jolla, CA) was used in all statistical analysis. Results of the toxicology experiments with gap junction inhibitors (i.e., adult hemolymph injections, adult topical applications, and larval assays) were analyzed using a non-linear curve fit analysis (log [inhibitor] vs. response variable-slope) to determine effective dose or concentration 50% values (i.e., ED_50_ or EC_50_). Results from the excretion assays were analyzed with a one-way ANOVA with a Newman-Keuls post hoc analysis. Relative gene expression was determined utilizing the delta CT method by normalizing target gene expression to that of the reference gene RPS7. Relative gene expression levels in eGFP controls were analyzed with a one-way ANOVA with a Newman-Keuls post hoc analysis. Percent gene silencing was calculated as in Drake et al. [24] by setting relative gene expression in eGFP injected mosquitoes to 100%. Significant knockdown was determined via Student’s t-tests comparing normalized innexin mRNA levels in eGFP dsRNA injected mosquitoes to that of innexin dsRNA injected mosquitoes. Survival between eGFP and innexin injected groups was compared by a two-way repeated measures ANOVA with Holm-Sidak’s post hoc analysis.

## Results

### Adult hemolymph injections

**Fig 1:**
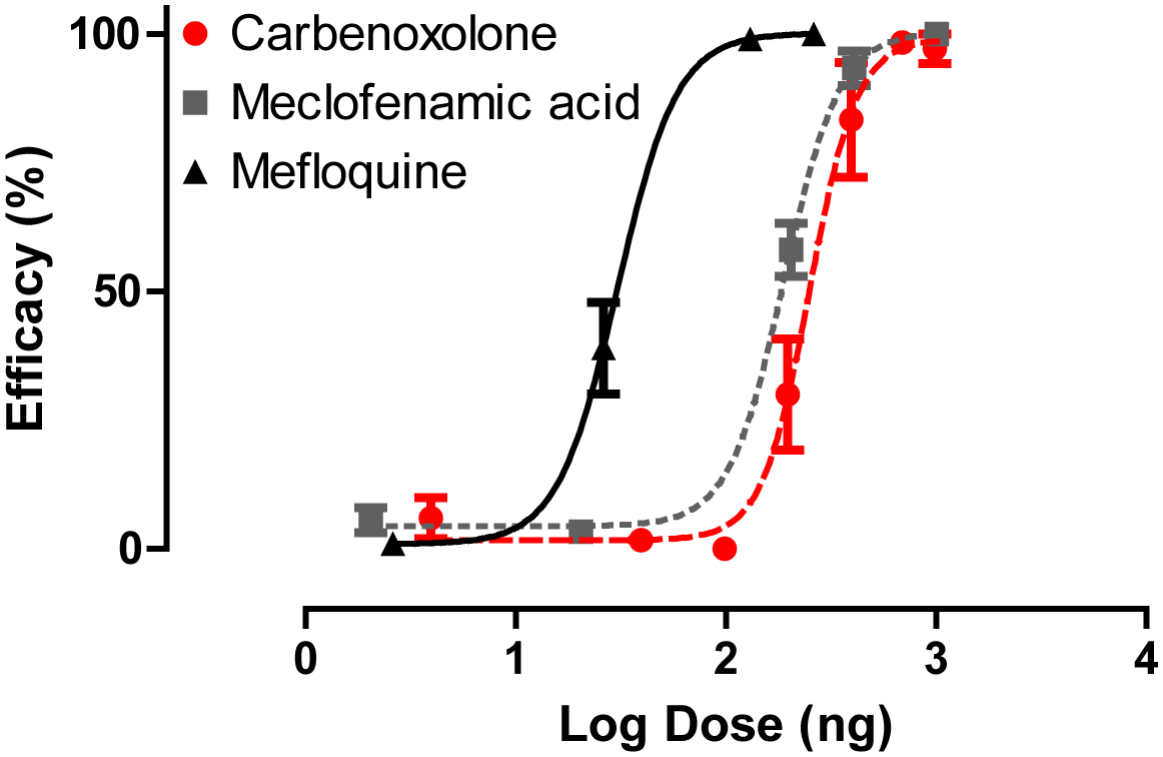
Dose-response curves of gap junction inhibitors injected directly into the hemolymph of adult female *A*. *aegypti* mosquitoes (carbenoxolone R^2^ = 0.873, meclofenamic acid R^2^ = 0.957 and mefloquine R^2^ = 0.906). Efficacy (dead and flightless mosquitoes) was assessed 24 h after injection. Taking into consideration the average mass of an adult female mosquito (1.97 mg), the ED_50_ for carbenoxolone, meclofenamic acid and mefloquine are 127.3 ng/mg, 96.4 ng/mg and 15.47 ng/mg respectively. Values are means ± SEM. n = 5–10 replicates of ten mosquitoes per dose tested.

To determine if gap junction inhibitors are toxic to adult female mosquitoes, we injected the inhibitors directly into the hemolymph. Carbenoxolone, mefloquine and meclofenamic acid all showed dose-dependent toxic effects in adult female mosquitoes (Fig 1). Mefloquine was the most effective inhibitor, with an effective dose for 50% of the population (ED_50_) of 15.47 ng per mg of mosquito body weight (ng/mg) followed by meclofenamic acid (ED_50_ = 96.39 ng/mg) and carbenoxolone (ED_50_ = 127.3 ng/mg; Fig 1).

### Adult topical assays

**Fig 2:**
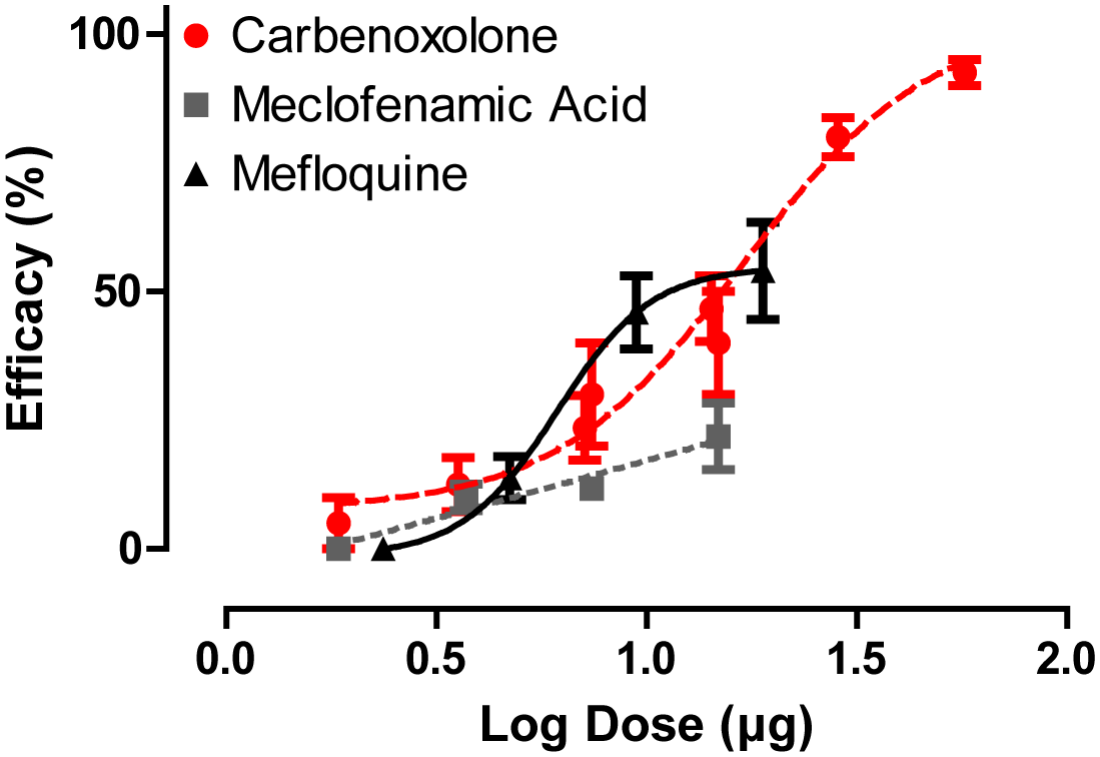
Dose-response curves of gap junction inhibitors applied directly to the cuticle of adult female *A*. *aegypti* mosquitoes (carbenoxolone R^2^ = 0.824, meclofenamic acid R^2^ = 0.455 and mefloquine R^2^ = 0.44). Efficacy (dead and flightless mosquitoes) was assessed 24 h after application. Taking into consideration the average mass of an adult female mosquito (1.97 mg), the ED_50_ for carbenoxolone is 8.57 μg/mg. The ED_50_ values for meclofenamic acid and mefloquine are not determinable. Values are means ± SEM. n = 4–8 replicates of ten mosquitoes per dose tested.

To determine whether the gap junction inhibitors can penetrate the cuticle, we evaluated the efficacy of the inhibitors in adult female mosquitoes when applied topically to the thorax. Of the three inhibitors, carbenoxolone was the only one to show a dose-dependent effect nearing 100% efficacy, with an ED_50_ of 8.57 μg/mg. Mefloquine showed limited dose-dependent effects, with a maximal efficacy of only ~54.5%. Meclofenamic acid showed the weakest topical efficacy (Fig 2).

### Adult excretion assays

**Fig 3:**
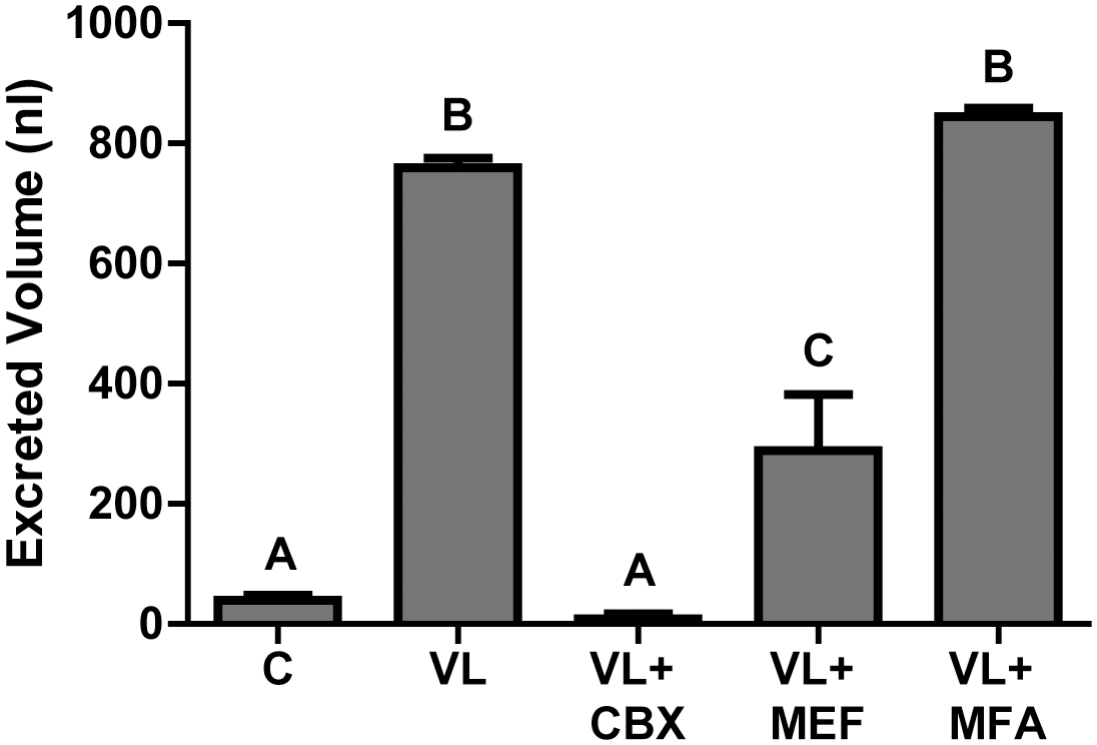
Effects of gap junction inhibitors on the diuretic capacity of adult female *A*. *aegypti* mosquitoes. C = non-injected control mosquitoes. VL = volume loaded mosquitoes injected with 900 nl of HBS. VL + CBX = mosquitoes injected with 900 nl of HBS and 1.34 mM carbenoxolone. VL + MEF = mosquitoes injected with 900 nl of HBS and 0.5 mM mefloquine. VL + MFA = mosquitoes injected with 900 nl of HBS and 1.53 mM meclofenamic acid. Letters (A, B or C) indicate statistical differences as determined by a one-way ANOVA and Newman-Keuls post-test (P < 0.05). Values are mean volumes of urine excreted per mosquito after two hours ± SEM. n = 14 for VL, n = 14 for C, n = 8 for VL + CBX, n = 6 for VL + MEF, and n = 6 for VL + MFA.

To determine if the gap junction inhibitors disrupt the diuretic capacity of adult female mosquitoes, we volume loaded their hemolymph with a sub-lethal dose of each inhibitor. Fig 3 shows the mean volume excreted per female in 2 h after injection for each treatment, compared to the non-injected controls (C). Mosquitoes injected with a volume load (VL) and no inhibitor excreted on average 760 ± 16 nl (Fig 3). In contrast, those injected with a VL and 1.34 mM carbenoxolone (VL + CBX) excreted a significantly lower amount of urine (8.0 ± 8 nl) that is similar to non-injected control mosquitoes, which excrete 39 ± 8 nl (Fig 3). Mosquitoes injected with a VL and 0.5 mM mefloquine (VL + MEF) excrete 290 ± 93 nl, which was significantly lower than the amount excreted by the VL mosquitoes, but significantly higher than that excreted by the VL + CBX mosquitoes (Fig 3). Mosquitoes injected with a VL and 1.53 mM meclofenamic acid (VL + MFA) excrete 844 ± 14 nl, which was comparable to that of VL mosquitoes (Fig 3). Concentrations of meclofenamic acid higher than 1.53 mM were lethal to the mosquitoes before the end of the 2 h excretion assay (data not shown).

### Larval assays

**Fig 4:**
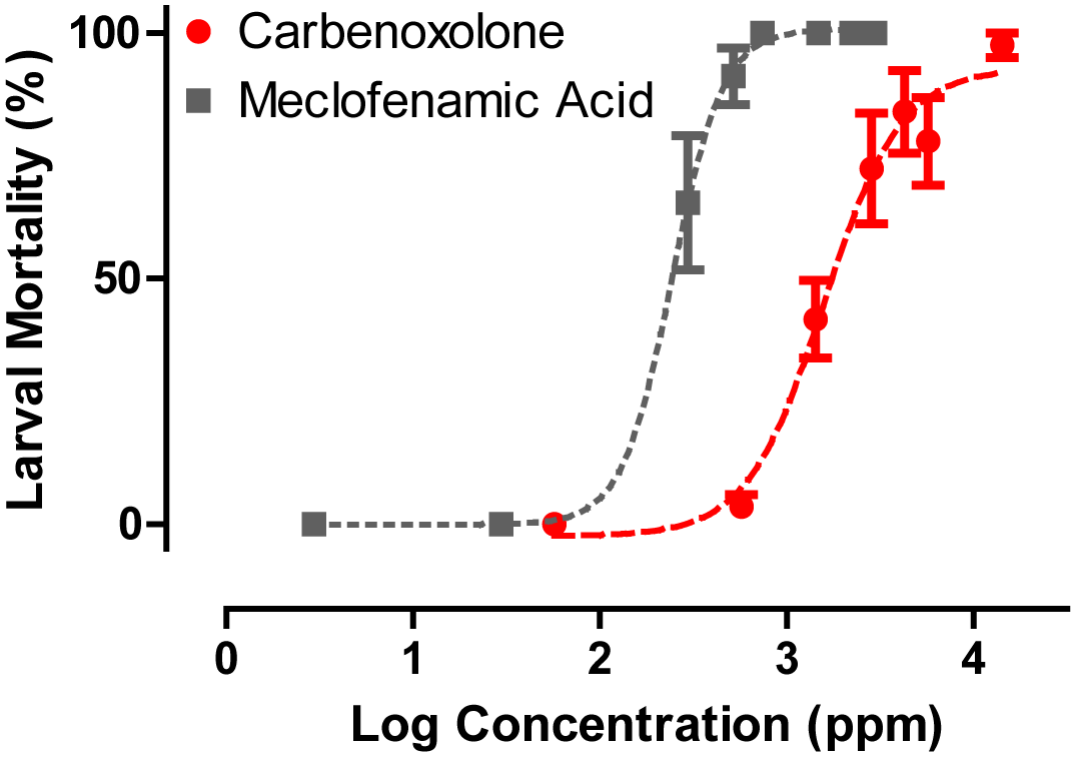
Dose-response curves of gap junction inhibitors added to the rearing water of 1^st^ instar larval *A*. *aegypti* mosquitoes (carbenoxolone R^2^ = 0.873 and meclofenamic acid R^2^ = 0.957). Larval mortality was assessed 24 h after adding the inhibitors. LC_50_ for meclofenamic acid and carbenoxolone are 0.83 mM and 2.84 mM, respectively. Values are means ± SEM. n = 4–8 replicates of five larvae per concentration tested.

We assessed the efficacy of the gap junction inhibitors as larvicides by adding them to the rearing water of 1^st^ instar larvae. Carbenoxolone and meclofenamic acid both showed dose-dependent toxic effects in larvae (Fig 4). Meclofenamic acid was the most effective (EC_50_ = 244.9 ppm) followed by carbenoxolone (EC_50_ = 1587 ppm). We were unable to test the efficacy of mefloquine in this assay, because it is not soluble in water and the amount of DMSO required to keep it in solution (>2%) was toxic to larvae.

### RNA interference (RNAi)

**Fig 5:**
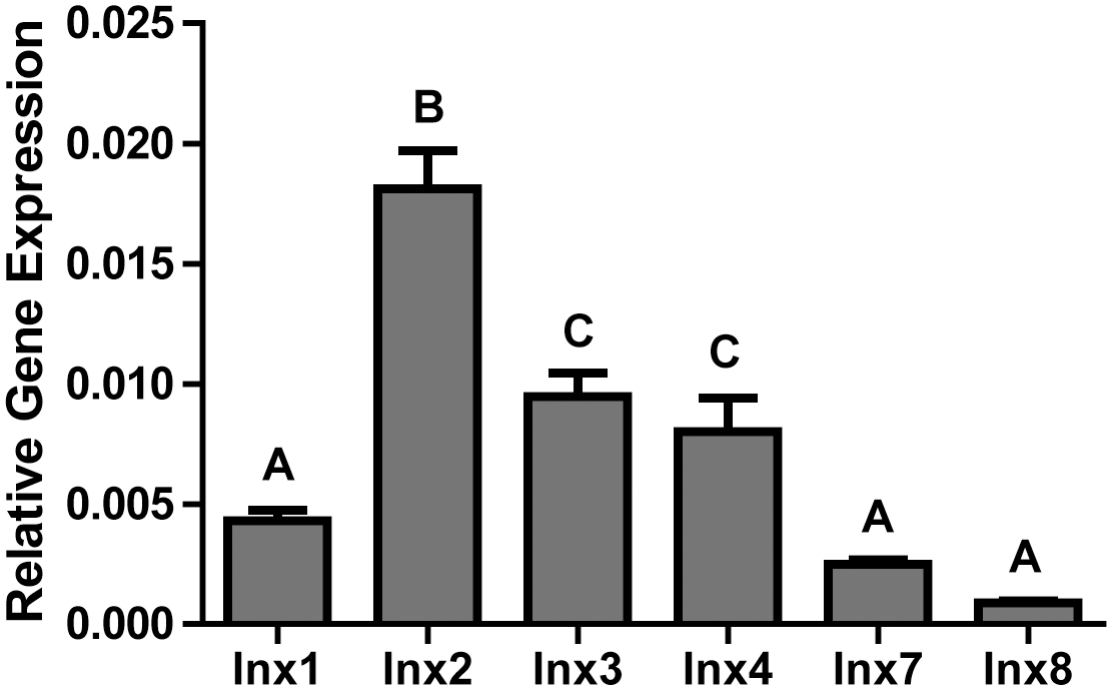
Relative innexin gene expression as normalized to RPS7 gene expression in eGFP dsRNA injected mosquitoes 3 days after injection. Values are means ± SEM. n = 4 replicates of 3 mosquitoes. Letters (A, B or C) indicate statistical differences as determined by a one-way ANOVA and Newman-Keuls post-test (P < 0.05).

To determine if knockdown of innexin mRNA levels affected the survival of adult female mosquitoes, we utilized RNAi. First, we used qPCR to determine the relative expression of each innexin in mosquitoes injected with eGFP dsRNA (3 days post injection). As shown in Fig 5, Inx2 was the most abundant innexin, followed by Inx3 and Inx 4. The expression of Inx1, Inx7 and Inx8 were lower, but detectable.

**Fig 6:**
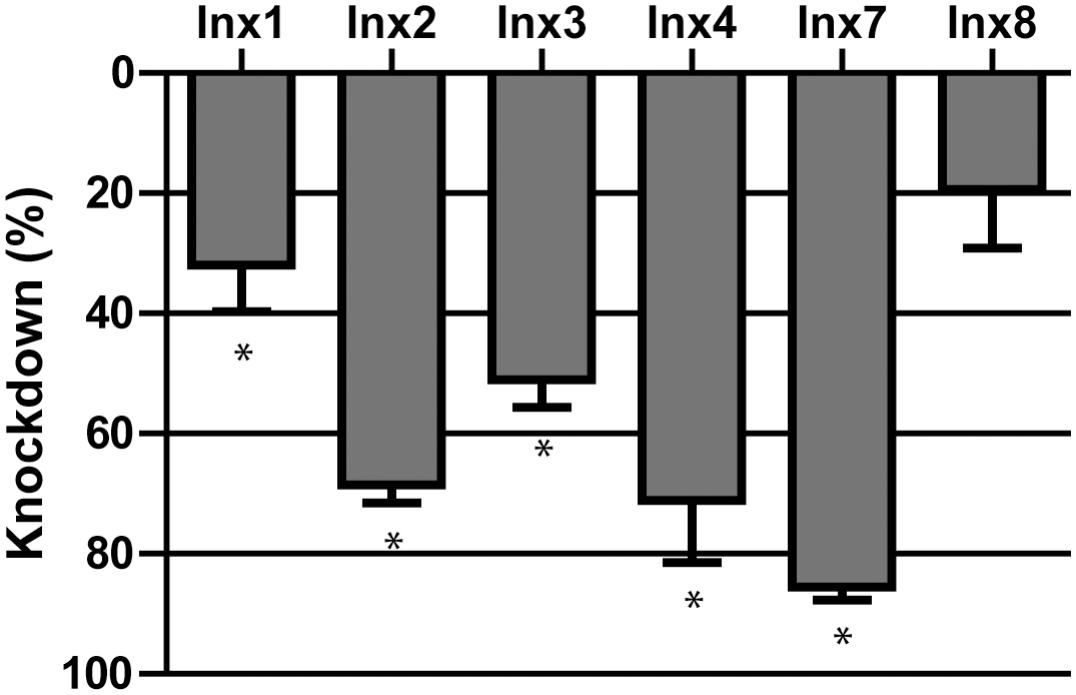
Knockdown efficiency in innexin dsRNA injected mosquitoes 3 days after dsRNA injection. Percent knockdown is relative to eGFP dsRNA injected control mosquitoes 3 days after injection (Fig 5). Values are means ± SEM. n = 4 replicates of 3 mosquitoes. Asterisks indicate significant knockdown compared to eGFP as determined by a Student’s t-test (p < 0.05).

**Fig 7:**
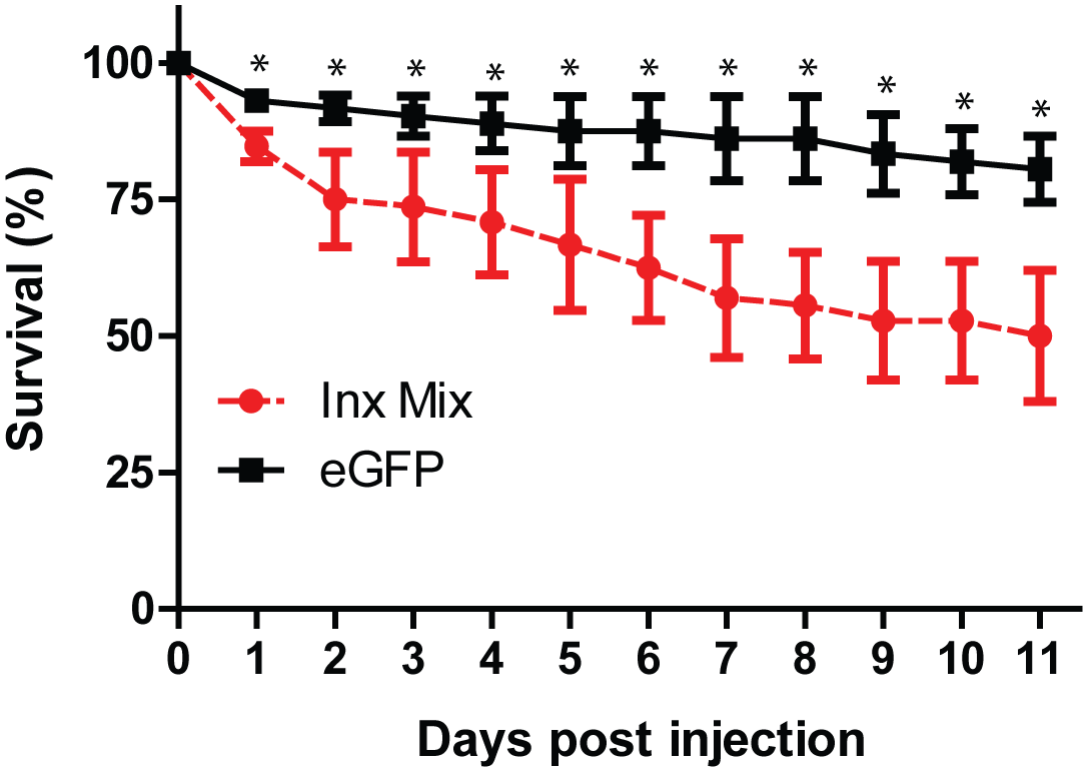
Effect of eGFP (black squares) and innexin (red circles) dsRNA injection on mosquito survival. Values are means ± SEM. n = 3 replicates of 24 mosquitoes. Asterisks indicate a significant difference in survival between eGFP and innexin dsRNA injected mosquitoes as determined by a two-way ANOVA with a Holm-Sidak’s post-hoc test.

Injection of an innexin dsRNA cocktail containing dsRNAs for each innexin (333 ng per innexin, 2 μg total) resulted in significant knockdown of Inx1 (32 ± 8%), Inx2 (69 ± 3%), Inx3 (51 ± 5%), Inx4 (71 ± 10%) and Inx7 (86 ± 2%) by 3 days after injection compared to expression levels in the eGFP-injected controls (Fig 6). The expression levels of Inx8 were not significantly knocked down, but the mRNA levels were very low to begin (Fig 5). The knockdown of innexin expression in mosquitoes injected with innexin dsRNA persisted until at least day 11 (data not shown). In addition, mosquitoes injected with innexin dsRNA exhibited a significantly lower survival than those injected with eGFP dsRNA that progressed over the next 11 days, and started as early as 1 day after injection (Fig 7).

## Discussion

Our study provides the first pharmacological and molecular evidence that suggests gap junctions are potentially valuable targets for mosquitocide discovery and development. When injected into the hemolymph of adult female mosquitoes, three structurally unique pharmacological agents that are known to inhibit gap junctions (carbenoxolone, meclofenamic acid, and mefloquine) were efficacious (Fig 1), and one of the compounds (carbenoxolone) exhibited the ability to penetrate the cuticle of adult females (Fig 2). Moreover, two of the gap junction inhibitors (carbenoxolone and meclofenamic acid) were effective when added to the rearing water of 1^st^ instar larvae (Fig 4). Thus, our data provide proof-of-concept that chemical inhibitors of gap junctions exhibit insecticidal properties in adult and larval mosquitoes.

In support of our pharmacological data, the injection of dsRNA against all 6 innexin mRNAs reduced the survival of adult female mosquitoes over the next 11 days (Fig 7). The reduced survival was associated with the significant knockdown of mRNA levels for 5 of the targeted innexins (Fig 6), which presumably leads to reductions of innexin protein levels and higher mortality. The mRNA levels of Inx8 were not significantly affected by dsRNA injection, but this gene is expressed at nominal levels in adult female mosquitoes (Fig 5) [11]. Notably, the degree and rate at which the toxic effects manifest via RNAi are respectively weaker and slower than those of the pharmacological inhibitors. The weaker effects of RNAi are likely attributable to a combination of the incomplete knockdown of innexin mRNA expression (Fig 6) and the time required for dsRNAs to elicit an effect on protein levels, which may lag behind that of mRNA levels. In contrast, pharmacological inhibition can be complete and elicit acute effects. Other studies have also noticed weaker toxic effects of inhibition of an insecticide target via RNAi vs. pharmacological inhibition [25–27].

The toxic effects of the gap junction inhibitors and the innexin dsRNAs on mosquitoes are not surprising given that innexin mRNAs are expressed throughout the mosquito life cycle [11]. Furthermore, in adult female mosquitoes, we have shown that at least 4 innexin mRNAs are expressed in each tissue of the alimentary canal (i.e., midgut, hindgut and Malpighian tubules), the ovaries, head, and thorax/abdomen [11]. Thus, the pharmacological or genetic inhibition of innexin function may cause disruptions to the nervous, digestive, excretory, and/or reproductive systems, leading to the impairment of flight and/or death. Further investigations will be required to confirm that such wide-spread disruptions to mosquito physiology are indeed occurring.

In the present study, we show that at least two of the pharmacological inhibitors (carbenoxolone and mefloquine) perturb the functions of the excretory system, as indicated by their inhibition of the diuretic capacity of adult female mosquitoes (Fig 3). These two inhibitors may be acting on the Malpighian tubules, which produce urine via transepithelial fluid secretion, and/or the hindgut, which attenuates the composition of urine before expelling it from the animal via muscular contractions. In Malpighian tubules, several lines of evidence suggest that gap junctions composed of innexins occur between the epithelial cells and play important roles in intercellular communication and diuresis [15,28–30]. Furthermore, in the hindgut, we have localized the expression of Inx3 immunoreactivity to the intercellular membranes of epithelial cells in the ileum and rectum [11]. Thus, inhibiting the activity of gap junctions in these tissues is expected to disrupt urine production and/or expulsion. However, additional experiments such as Ramsay assays of isolated Malpighian tubules and isolated hindgut contraction assays [31,32] are needed to resolve the mechanisms by which carbenoxolone and mefloquine inhibit excretory performance.

Surprisingly, we did not observe effects of a sub-lethal dose of meclofenamic acid (1.53 mM) on the diuretic capacity of mosquitoes (Fig 3), and higher doses were lethal before the 2 hr experimental period finished (Calkins, unpublished observations). These findings suggest that meclofenamic acid either does not inhibit gap junctions expressed in the excretory system of mosquitoes, or it may elicit rapid toxic effects elsewhere, such as in the nervous system before any effects on excretory function can be observed. Perhaps, meclofenamic acid is able to cross the blood-brain barrier of mosquitoes more efficiently than carbenoxolone and mefloquine, thereby leading to a more rapid toxic effect.

### Potential for gap junction inhibitors as insecticides

**Fig 8:**
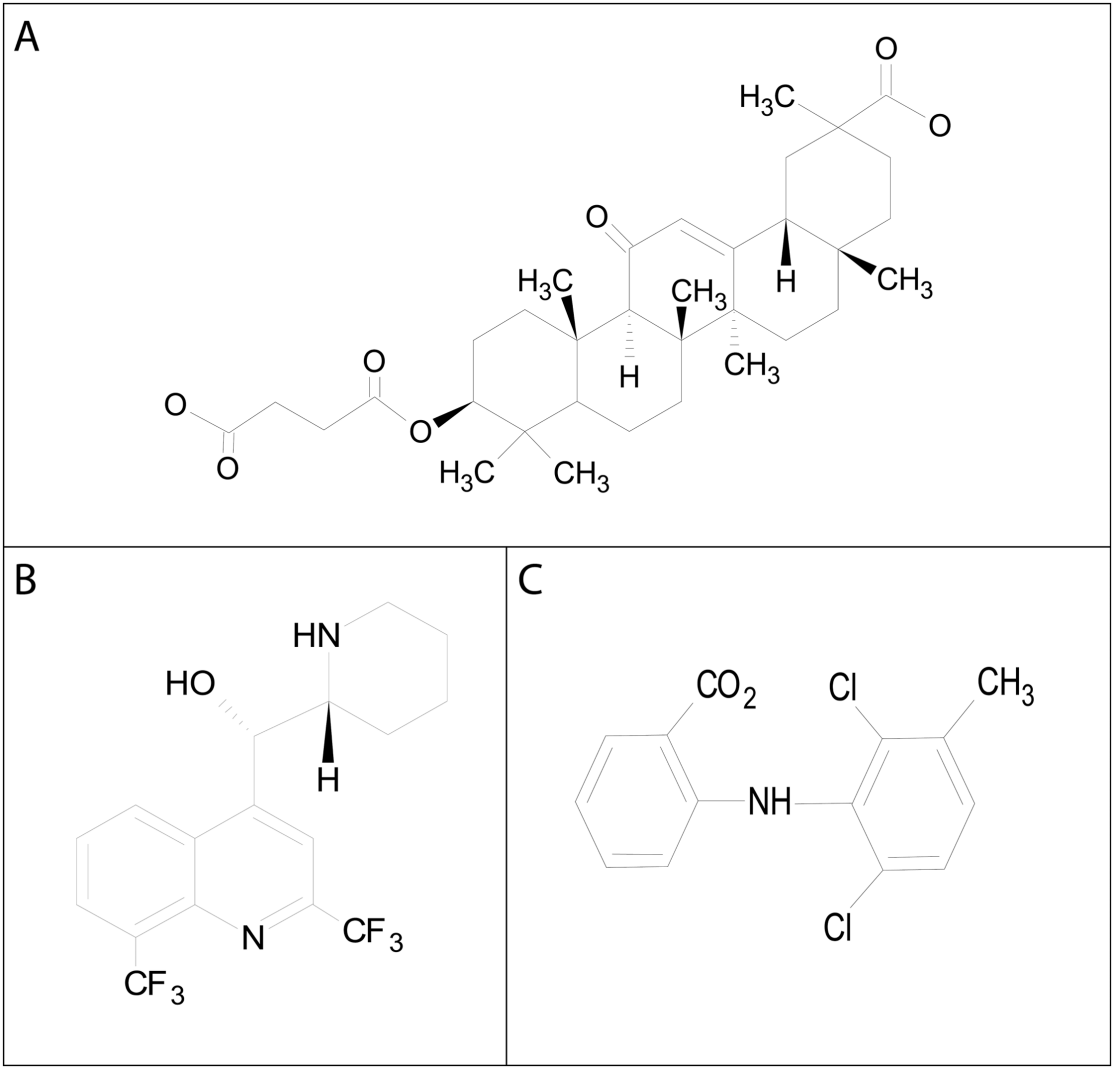
Molecular structures of the gap junction inhibitors: A) carbenoxolone, B) mefloquine, and C) meclofenamic acid. Structures as shown were modified from Sigma-Aldrich (www.sigma.com) and constructed in ChemSketch (ACD/Labs, Toronto, Ontario, Canada).

When applied topically to the cuticle of mosquitoes, only carbenoxolone showed insecticidal activity, whereas mefloquine and meclofenamic acid had limited and nominal activity, respectively. Thus, carbenoxolone appears to have the capacity to penetrate the cuticle. The structures (Fig 8) and chemical properties (Table 3) of these three gap junction inhibitors are distinct and may explain their different abilities to penetrate the cuticle. For example, when evaluating the three inhibitors based on the “Rule of 5” [33,34] for identifying insecticides (Table 3), carbenoxolone adheres closely with a ‘clog p’ below 5, zero hydrogen bond donors, 7 hydrogen bond acceptors, only 6 rotatable bonds, and a molecular weight near 500 kDa (Table 3). Besides molecular weight, the other two gap junction inhibitors differ from carbenoxolone primarily in their number of hydrogen bond donors. Carbenoxolone is the only one of the three compounds that adheres to the norm of current insecticides [33] with no hydrogen bond donors.

**Table 3:** Molecular properties of the gap junction inhibitors (rows 2–4) as compared to the properties identified by Tice [33] for screening for novel insecticides (row 5).

Inhibitor	Molecular Weight	cLog P	Hydrogen Bond Donors	Hydrogen Bond Acceptors	Rotatable Bonds
Carbenoxolone	570.77	4.83	0	7	6
Mefloquine	378.32	3.70	2	3	4
Meclofenamic Acid	296.15	4.92	2	3	3
**Tice Rule of 5**	**< 500**	**< 5**	**0**	**< 10**	**< 12**

Despite nominal topical efficacy against adult female mosquitoes, meclofenamic acid was the most potent larvicide of the three compounds (Fig 4). Carbenoxolone was also effective against larvae, but over 6-fold less potent than meclofenamic acid. Given that meclofenamic acid was unable to penetrate the cuticle of adult females (Fig 2), we presume that in larvae this compound is delivered to the alimentary canal via ingestion where it may act on the midgut epithelium and/or diffuse into the hemolymph.

Although meclofenamic acid and carbenoxolone were respectively the most effective compounds against larval and adult female (topically) mosquitoes, it is important to emphasize that these compounds are not nearly as potent as conventional insecticides, such as permethrin. For example, meclofenamic acid is 46 times less effective against 1^st^ instar larvae of *A*. *aegypti* than DNOC (Dinitro-ortho-cresol), which is considered a weak insecticide, and 874,643 times less effective than permethrin, a highly potent insecticide [22]. Additionally, our most topically active inhibitor, carbenoxolone, is 5.7 times less effective than bifenzate, a very weak mosquitocide and over 18.5 million times less effective than fipronil, the most potent adulticide against *A*. *aegypti* [22].

Additional concerns for both carbenoxolone and meclofenamic acid are their efficacy on mammalian gap junctions [35,36]. Moreover, carbenoxylone has potential off target effects in mammals, and meclofenamic acid, is currently used therapeutically as a nonsteroidal anti-inflammatory drug (NSAID) pain reliever [36,37]. Thus, our data should only be taken as proof-of-concept that gap junction inhibitors possess insecticidal properties. Additional efforts will be necessary to modify the potency and selectivity of these compounds for mosquitoes before they could be considered for use as insecticides in the field.

## Conclusions

The present study is the first to demonstrate that three different commercially available gap junction inhibitors exhibit insecticidal activity. Moreover, we show that RNAi-based knockdown of mRNAs encoding gap junctional proteins (i.e., innexins) leads to increased mortality of adult female mosquitoes. Taken together, these results suggest that gap junctions offer new potential insecticidal targets for mosquito control. However, much progress still needs to be made in terms of discovering compounds with acceptable potency and specificity for mosquito gap junctions. The long evolutionary distance between innexins and connexins leaves us hopeful that an innexin-specific compound can be discovered and developed.
